# Impact of rotation direction on treating common bile duct stones with an asymmetrical 8-wire helical basket

**DOI:** 10.1055/a-2589-1302

**Published:** 2025-05-22

**Authors:** Mamoru Takenaka, Tomohiro Fukunaga, Atsushi Nakai, Shunsuke Omoto, Chang-Il Kwon, Masatoshi Kudo

**Affiliations:** 1 38158 Department of Gastroenterology and Hepatology, Kindai University Faculty of Medicine Graduate School of Medical Sciences, Osakasayama, Japan; 2 37129 Digestive Disease Center, CHA Bundang Medical Center, Seongnam, Republic of Korea

In recent years, 8-wire helical basket catheters have emerged as effective tools for retrieving small common bile duct stones (CBDSs) measuring ≤10 mm 1 2 3 . Notably, the novel nitinol 8-wire helical basket catheter with a rotation function, the RASEN2 (Kaneka Corporation), has shown promising results 4 5 . A key feature of this basket is its asymmetrical shape, enabling clockwise or counterclockwise rotation ( Fig. 1 ). However, the impact of rotation direction on the treatment of small CBDSs and debris has not been investigated.

**Fig. 1:**
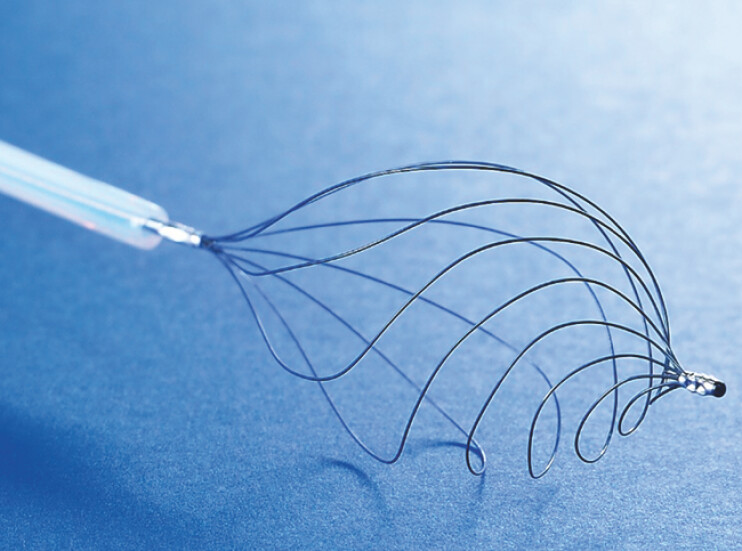
A novel nitinol 8-wire helical basket catheter with a rotation function (RASEN2; Kaneka Corporation). Its key feature is its asymmetrical shape, which can be rotated in the clockwise or counterclockwise direction.

In vitro experiments using the bending bile duct with corner pockets model ( Fig. 2 ) revealed that counterclockwise rotation exhibited superior efficacy in removing debris and stones. While sweeping with clockwise rotation, debris was scraped out by the basket; however, residual debris was significant. On the other hand, during counterclockwise sweeps, debris scraped by the basket wires was observed to collect and accumulate within the basket lumen, facilitating en masse removal ( Fig. 3 ). Although a balloon catheter could release stones lodged in the bile duct flexure and corner pockets, the 8-wire helical basket with a counterclockwise rotation demonstrated the ability to retrieve lodged stones even from the corner pocket of the CBD terminus ( Fig. 4 ).

**Fig. 2:**
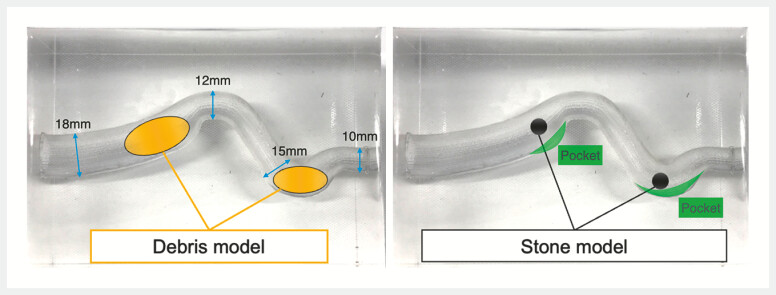
The bending bile duct with corner pockets model.

**Fig. 3:**
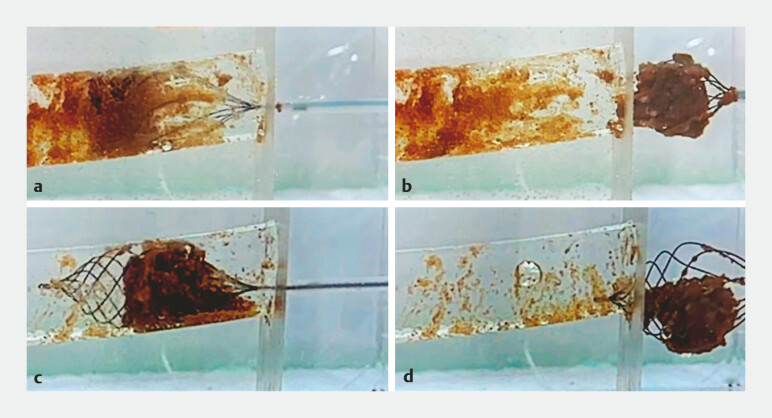
**a, b** While sweeping with clockwise rotation, debris was scraped out by the basket; however, residual debris was significant. **c, d** While sweeping with counterclockwise rotation, debris scraped out by the basket wire accumulated within the basket lumen, showing en masse removal.

**Fig. 4:**
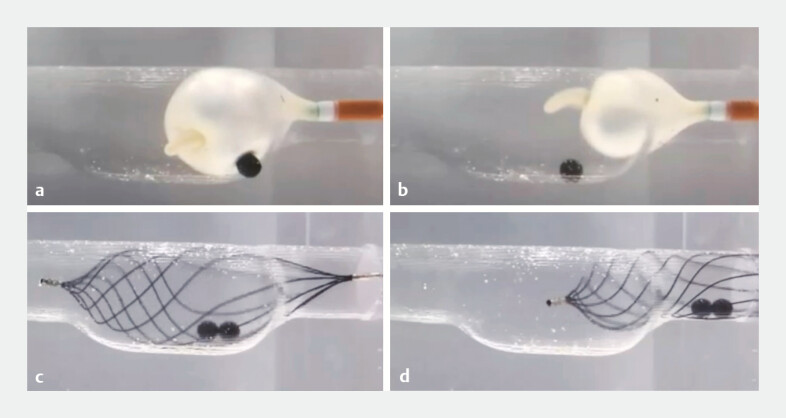
**a, b** The balloon catheter released the stones in the bile duct flexure and the corner pocket of the CBD terminus. **c, d** An 8-wire helical basket sweep with counterclockwise rotation could remove the stones, even those lodged within the corner pocket of the CBD terminus. Abbreviation: CBD, common bile duct stone.

A 58-year-old patient with small CBDSs underwent endoscopic treatment using RASEN2.

After repeated attempts to remove the stones using clockwise rotation, it was judged that no further stones could be captured. In contrast, subsequent sweeps with counterclockwise rotation yielded residual stones that were not retrieved with initial sweeps using clockwise rotation ( Fig. 5 , Video 1 ). The patient has not experienced recurrent CBDSs since the procedure.

**Fig. 5:**
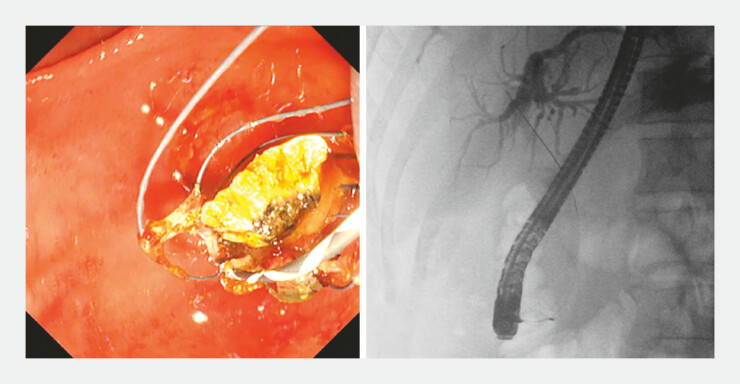
Subsequent sweeps with anticlockwise rotation to check for residual stones yielded stones that were missed during the initial clockwise rotation sweeps.

This video shows the impact of rotation direction on the treatment of common bile duct stones using an asymmetrical 8-wire helical basket.Video 1

These findings underscore the significant impact of rotation direction on the treatment of CBDSs using the asymmetrical-shaped 8-wire helical basket with a rotation function.

Endoscopy_UCTN_Code_TTT_1AR_2AH

## Notes

Endoscopy E-Videos https://eref.thieme.de/e-videos
*E-Videos* is an open access online section of the journal *Endoscopy* , reporting on interesting cases and new techniques in gastroenterological endoscopy. All papers include a high-quality video and are published with a Creative Commons CC-BY license. Endoscopy E-Videos qualify for HINARI discounts and waivers and eligibility is automatically checked during the submission process. We grant 100% waivers to articles whose corresponding authors are based in Group A countries and 50% waivers to those who are based in Group B countries as classified by Research4Life (see: https://www.research4life.org/access/eligibility/ ). This section has its own submission website at https://mc.manuscriptcentral.com/e-videos .
